# Assessment of the influence of lower limb cycling on arm muscle coordination during upper limb cycling: a pilot study

**DOI:** 10.1186/s12984-026-01959-y

**Published:** 2026-03-28

**Authors:** Lilla Botzheim, Balazs Radeleczki, Mariann Mravcsik, Jose L. Pons, Filipe Oliveira Barroso, Jozsef Laczko

**Affiliations:** 1 Neurorehabilitation and Motor Control Research Group, Department of Computational Sciences, HUN-REN Wigner Research Centre for Physics, Budapest, Hungary; 2 Faculty of Science, University of Pecs, Pecs, Hungary; 3 Rehabilitation Clinic, Semmelweis University, Budapest, Hungary; 4 Faculty of Information Technology and Bionics, Pazmany Peter Catholic University, Budapest, Hungary; 5 Legs & Walking Lab, Shirley Ryan AbilityLab, Chicago, IL USA; 6 Department Physical Medicine and Rehabilitation, Northwestern University, Chicago, IL USA; 7 Neural Engineering Lab, Cajal Institute, Spanish National Research Council, Madrid, Spain

**Keywords:** Muscle synergies, Arm cycling, Cycling mode

## Abstract

**Introduction:**

Arm cycling and leg cycling are motor tasks commonly used in medical rehabilitation. Although there is agreement on the existence of common neural generators between arm and leg cyclic movements, most studies have assessed the effects of upper limb cycling on lower limb activation. This study aimed to investigate whether leg cycling, added simultaneously to arm cycling induces changes in upper limb muscle coordination. The muscle synergy framework was used for the examination.

**Methods:**

Ten able-bodied women performed cycling trials on an arm and leg cycle ergometer, with two different cycling modes: a) only arm cycling, b) cycling with arms and legs simultaneously. In each cycling mode, the arms cycled against two crank resistances (low and moderate), whereas the resistance of the cranks for the legs was always low. For each combination of cycling mode and resistance (cycling setup), participants cycled for three minutes, and electromyography signals from six arm muscles from each side were recorded. For each cycling setup, muscle synergy analysis was carried out, using a non-negative matrix factorization algorithm.

**Results:**

Four synergies accounted for more than 90% of muscle activation variance in all the setups. Overall, arm muscle synergies were not affected by leg cycling and by the addition of arm crank resistance.

**Discussion:**

These results suggest that the central nervous system applies similar control strategies for arm cycling regardless of the inclusion of leg cycling. These findings enhance our understanding of the motor control mechanisms of arm cycling. This is probably the first study investigating motor control of arm cycling during simultaneous arm and leg cycling.

## Introduction

Humans can produce a variety of rhythmic movement patterns, such as walking, running, swimming and cycling, among other forms of terrestrial and aquatic locomotion. Scientific evidence supports shared neural control across these movements. The study of Zehr et al. [1] supported the existence of common neural patterning as the regulator of arm and leg movements during some of these human rhythmic movements, suggesting coupling between the upper and lower limbs. Likewise, Weersink et al. [2] reported significant intermuscular coherence of arm and leg muscles in the alpha and beta/gamma bands during normal walking, providing additional evidence of shared neural control of arm and leg movements. The study of Wannier et al. [3] extends the concept of interlimb coordination to non-terrestrial movements, demonstrating that the arm to leg coordination observed in human walking also occurs during other human locomotor activities, such as swimming, swimming with flippers, and creeping on all fours as well. Other studies have assessed how arm cycling can modulate H-reflexes in leg muscles and how leg cycling can affect H-reflexes in arm muscles. Hundza et al. [4] demonstrated that active arm cycling can significantly suppress the soleus H-reflex amplitude, while Nakajima et al. [5] showed that leg cycling reduces the flexor carpi radialis H-reflex amplitude. A significant reduction in the amplitude of flexor carpi radialis H-reflexes during dynamic cycling of the legs was also found in neurologically intact participants by Zhou and colleagues [6]. This finding may indicate that cycling with the lower limbs can inhibit upper limb motoneurons, and this inhibition is mediated by cervico-lumbar pathways. These results support a conservation of neural control mechanisms between arms and legs during locomotor tasks in humans [5].

Although there is an agreement on the existence of common neural generators between arm and leg cyclic movements, most of the studies have assessed the effects of upper limb cycling or combined upper and lower limb cycling on lower limb activation [7–9]. The extent to which rhythmic lower limb training activates interlimb networks for upper limb cycling remains mostly unclear. Demonstrating that leg cycling or combined arm and leg cycling changes the activation of upper limb muscles can have translational implications for rehabilitation where each of these activities could be applied to improve upper limb function. To the best of our knowledge, only Weersink et al. [2] assessed this through directed connectivity analyses, with results suggesting that upper limb muscles drive and shape lower limb muscle activity during gait via subcortical and cortical pathways and, to a lesser extent, vice versa.

If lower limb cycling does not affect arm muscle coordination, that suggests that the regulation of arm muscle activities may not be perturbed or affected by movements of the lower limbs. On the other hand, perturbation of normal lower limb activities or tasks has been shown to affect arm muscle activities. For instance, adding a load to the ankle during walking produced increased muscle activity and movement amplitude in both arms [10, 11]. Sakamoto et al. [12] reported that during simultaneous arm and leg cycling, the change of cadence of leg cycling influenced the cadence of arm cycling, indicating that leg cycling affects arm cycling during simultaneous arm and leg cycling.

The main objective of our study was to test the hypothesis that leg cycling, added simultaneously to arm cycling affects arm muscle coordination, and that addition of arm crank resistance alters arm muscle synergies components during both arm-only cycling and simultaneous arms and leg cycling. To assess muscle coordination, we used the analysis of muscle synergies.

The above cited papers investigated the relationship between kinematic data, reflexes (e.g., H-reflexes) and electromyographic activity of individual muscles. While these studies have made valuable contributions to our understanding of the motor control of cyclic upper and lower limb movements, they address different aspects of motor control than muscle synergy analysis and muscle coordination. Muscle synergies provide a mathematical framework in time domain to model the active, voluntary motor control strategies of the central nervous system, describing the simultaneous coordination of multiple muscles. The concept of muscle synergies suggests that the central nervous system (CNS) sends neural commands to activate specific muscle groups to produce movement instead of activating muscles individually to achieve the same functional task [13]. Muscle synergies are widely employed in both motor control research and clinical applications [14, 15]. Utilizing this framework allows the interpretation of our findings within a broader functional and rehabilitative context. Understanding muscle coordination in healthy participants is essential for developing customized rehabilitation approaches, which often include upper limb cycling.

 The analysis of muscle synergies has previously been carried out by us during lower limb cycling [16] and during upper limb cycling [17]​ separately. Independently from our study, Cartier et al. compared the muscle synergies during separately performed upper and lower limb cycling [18] and also investigated the muscle synergies during only arm cycling [19]. However, to the best of our knowledge, muscle synergies have not yet been investigated during simultaneous arm and leg cycling.

## Methods

### Participants

Ten healthy women (age 22.2 ± 3.7 years, height 1.64 ± 0.06 m, weight 59.6 ± 5.3 kg) without any known orthopedic or neurological problems participated in this study and provided informed consent. All of them were right-handed and right-footed. The experiments were conducted in accordance with the Declaration of Helsinki. The Ethics Committee of the National Medical Institute for Rehabilitation, Budapest, Hungary (presently Semmelweis University, Rehabilitation Clinic) provided approval for this research (protocol number: 20/2017/10/04) and all participants provided informed consent.

### Experimental setup and procedures

**Fig. 1:**
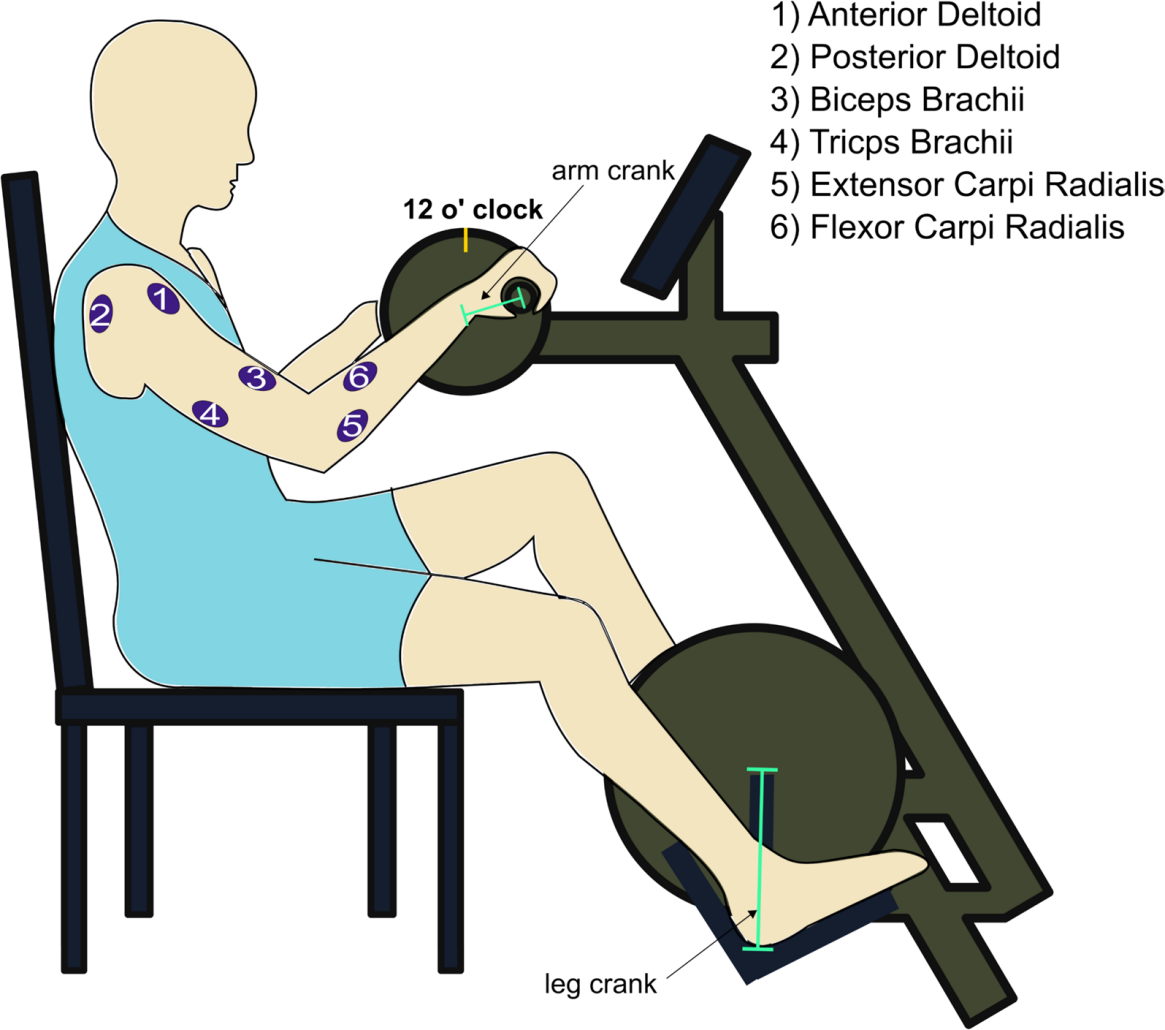
Example of a participant sitting in front of the ergometer while performing a cycling task. The electromyography signals from six arm muscles were recorded independently for the right and left sides

Each participant performed several cycling tasks on a MotoMed Viva2 arm and leg cycle ergometer (Reck GMBH, Betzenweiler, Germany). A schematic figure of the arm and leg cycle ergometer and a cycling participant is presented in Fig. 1. Each participant cycled with her arms against 2 different levels of arm crank resistance: low and moderate (level ‘5’ and level ‘10’ on the Motomed Viva2 cycle-ergometer), that corresponded approximately to 9 and 19 Watt power outputs in the two resistance levels. The motor of the lower cranks was switched off, so we used the passive resistance of the lower crank. Moreover, cycling was performed in 2 different modes: cycling only with the arms (“only-arm” mode) and cycling with both arms and legs (“arm&leg” mode). Therefore, each participant performed 4 different combinations of cycling: 2 cycling modes with 2 levels of resistance.

In the arm&leg cycling mode, each participant was asked to cycle with both arms and legs simultaneously, with the right arm cycling at the same cycling frequency and same phase of the left leg and to try to keep it synchronized during performing the arm&leg cycling task. For both the arm and the leg, the left crank and the right crank were mechanically coupled and were set 180 degrees out of phase, but the arm cranks were not coupled with the leg cranks. The arm cranks’ axis was set at the chest center level. Each participant was positioned as far as possible from the ergometer, guaranteeing that they were able to stretch their legs almost fully, similar to our previous work [20].

For each combination of cycling, surface electromyography (EMG) signals from six arm muscles were recorded for each side independently. First each participant performed each combination of cycling recorded from one side, and after a break, each combination was repeated, while data were recorded from the other side. The recorded muscles were the following: anterior deltoid (AD), posterior deltoid (PD), biceps brachii (BB), triceps brachii (TB), extensor carpi radialis (ECR), and flexor carpi radialis (FCR). A Cometa MiniWave (Cometa, Italy, Bareggio) wireless EMG system was used for the EMG recordings, with a sampling frequency of 1200 Hz. Electrode placement and skin surface preparation were carried out following SENIAM recommendations [21]. Simultaneously with EMG recording, an infrared (IR) six-camera motion analyzer system (Vicon Nexus, England, Oxford) was used to record arm crank movements.

Infrared reflective markers were placed on the lateral side of the arm cranks’ handles and of the pedals of the ergometer, and 3D coordinates of these markers were recorded with a sampling frequency of 120 Hz. Kinematic data were used to monitor and cut the data into cycles. At the beginning of the assessment, the order of the only-arm and arm&leg cycling modes and the order of the measured sides (left and right) were randomly selected. Moreover, the order of the resistance levels was as follows: low, moderate. This restriction was applied because, in arm&leg cycling, it was rather challenging for participants to adapt to different randomly selected resistance levels. In total, 8 setups were produced: 4 combinations from 2 sides. In each setup the participants had opportunity to try out the cycling modes before each recording. They got the instruction to start to execute the actual cycling task, and we gave them the opportunity to practice several dozens of cycles before we started the recording. A predefined time or cycle limit was not set; the recording started when the participant confirmed that a stable rhythm was achieved and the examiner also confirmed it by visual observation. The cycling frequency was predefined as 60 revolutions per minute (rpm). A 60-rpm audio feedback was provided by a metronome, which helped the participants to maintain the target cadence. For each setup, the participants cycled in total for three minutes (recorded in two or three intervals to reduce the possible measurement errors). Short breaks (60 s) were taken between these 3-min cycling trials to avoid muscle fatigue. A longer break (20 min) was also taken when the EMG recorders were placed from one side to the other (with new gel electrodes).

### Data processing

#### Cycles selection

The vertical coordinates of the marker on the right handle of the ergometer were used for cycle segmentation. One cycle was defined as an interval between two consecutive 12 o’clock positions of the right crank. These positions were applied for both arms, for the right and left too.

All the recorded EMG signals from each setup were band-pass filtered (25–300 Hz, 3rd-order Butterworth). We then applied a 2nd-order IIR notch filter at 50 Hz to these filtered data to remove power-line noise. The mean signal value was subsequently subtracted from the EMG signals. Finally, the EMG signals were rectified and low-pass filtered at 5 Hz (3rd-order Butterworth) to obtain the EMG envelopes [22, 23]. EMG envelopes were then time-normalized by resampling the data at each percentage of the cycle (101 points: 0–100).

EMG envelopes were analyzed on a cycle-by-cycle basis to identify outliers that needed to be removed. Within the total three minutes of cycling, there were segments, where measurement artifacts such as electrode contact failure were occurred, therefore these were discarded. Another type of artifacts such as break of the cycling or sudden phase shift were caused by the attentional shifts of the participants, since these exercises demand focused motor coordination. To decide whether a cycle was an outlier or not, we started by calculating the mean cycle and defined two thresholds. First, we assessed whether the mean cycle and each individual cycle were statistically correlated (correlation coefficient was greater than 0.3, *p* > 0.05, based on Student table). Second, we assessed whether the range of the amplitude (difference between the maximum and minimum) of each cycle was within the range of 50%–150% of the mean cycle. Another criterion was added: the retained data had to be at least 40,000 samples long (having a 1200 Hz sampling frequency, this means that for one setup, approximately at least 33 cycles were taken). Based on our previous work, these number of cycles is sufficient for investigating arm cycling tasks [23]. This criterion was used to retain cycles that were less spoiled by artifacts but still to achieve a meaningful number of cycles. If this criterion was not met, the threshold of the amplitude range was increased by ± 5%. Finally, the EMG envelopes corresponding to the selected cycles were concatenated for each subject, cycling mode and resistance. On average, more than 60 cycles were retained for further analysis (across participants) for each setup. This number of cycles is considered adequate, based on the recommendation of Turpin et al. [24].

For each participant [10], the maximum of EMG envelopes of all cycles for each setup [8] and muscle [6] were found. Then for each muscle, the largest one of these 8 EMG maximum values was used to normalize all EMG time series for that muscle.

#### Extraction of muscle synergies

For each setup, concatenated EMG envelopes were combined into *m* × *t* (EMG_0_) matrices, where *m* is the number of muscles (six, in this case) and *t* is the time base [number of concatenated cycles × 101]. Muscle synergies components were calculated applying a non-negative matrix factorization (NNMF) method [25] with MATLAB (MathWorks, Natick, MA, USA). Mathematically, the algorithm is described as

$$ EMG_{0} = \, WH \, + \, e \, = \, EMG_{r} + \, e $$

where *W* is the *m* × *n* matrix specifying the weight of each muscle in each synergy, *n* is the number of muscle synergies, and *H* is the *n* × *t* matrix specifying the time-varying activation coefficients representing the recruitment of each synergy throughout the cycle. EMG_*r*_ is the matrix *m* × *t* resulting from the multiplication of *W* and *H* (reconstructed EMG envelopes), and *e* is the residual error. We considered 3 and 4 synergies (*n* = 3, 4) as input to the NNMF algorithm. For each *n*, NNMF was run 40 times, and the repetition with the smallest reconstruction error was selected. The minimum number of synergies required to guarantee an adequate reconstruction of the EMG signals was determined as the minimum number necessary to obtain a variability accounted for (VAF) greater than or equal to 90% [16, 23, 26]. Based on these studies, the mean total VAF value was calculated and presented in the current work.

#### Synergy vectors ordering

We applied max-normalization on the W synergy weight matrix [27]. We normalized each column of the W synergy matrix by the maximal value of the column. Synergy vectors (columns of W matrices) were then ordered for each participant and setup, based on their similarity to each other. We used the cosine similarity of the synergy weights as a metric of similarity[16, 23]. For each setup, we ordered the synergy vectors of each participant compared to a reference participant. Next, for each setup, the corresponding synergy vectors were averaged across participants. Finally, we also ordered the averaged synergy vectors with respect to the reference setup (only arm cycling mode, low resistance, left side). The activation coefficients (H matrices) followed the order corresponding to the synergy vectors.

#### Quantitative comparison

Our main goal was to explore the effects of different cycling combinations (resistance or mode) on muscle synergies. First, the normality of the synergy vectors was checked with Shapiro–Wilk test. Given that the data did not show a normal distribution, non-parametric statistical methods were used. The elements of the synergy vectors, i.e., the activation coefficients and the synergy weights, were analyzed with several different methods to strengthen our findings.

The activation coefficients were averaged across cycles for each participant and time normalized to 101 samples (0–100). The averaged activation coefficient array for each cycling setup was created as follow: number of participants x number of synergies x length of averaged cycle (10 × 4 × 101).

To examine the similarity between the different cycling combinations, Spearman rank test was applied on the averaged cycle of activation coefficients in participants, separately the four coefficients. To calculate the significancy (*p*-values) of the correlation and the effect size (Cohen’s d), we applied Fisher-z transformation.

Moreover, we investigated also the differences between the cycling combinations on the mean activation coefficients. Statistical Parametric Mapping (SPM) vector-field analysis is an established method for quantitative correlation of biological time series [28]. This method is applicable to the statistical association of continuous biomechanical datasets, while numerous statistical tests (parametric and non-parametric) can be used within SPM. For pairwise comparisons of the activation coefficients, non-parametric paired T-test was applied within the SPM with Bonferroni correction. We used the SPM library for MATLAB, which is public.

The synergy weights were also compared between the different cycling combinations. To analyze the similarity of the different cycling combinations in terms of synergy weights, cosine similarity was calculated between the averaged (across participants) synergy weights [29].

We also investigated the differences between the synergy weights in cycling combinations, applying Friedman test for the synergy weights of each muscle in the JASP statistical software [30]. Conover post-hoc test was used [31], which is recommended after the Friedman test for pairwise comparisons [32]. The significance level was set at a p value of 0.05, with the application of the Holm-Bonferroni correction.

## Results

We compared the average EMG envelopes of the only-arm and arm&leg cycling exercises across participants. The total VAF values obtained were also investigated, and the resulting synergy weights (W) and coefficients (H) were investigated.

### Comparison of muscle activities

**Fig. 2:**
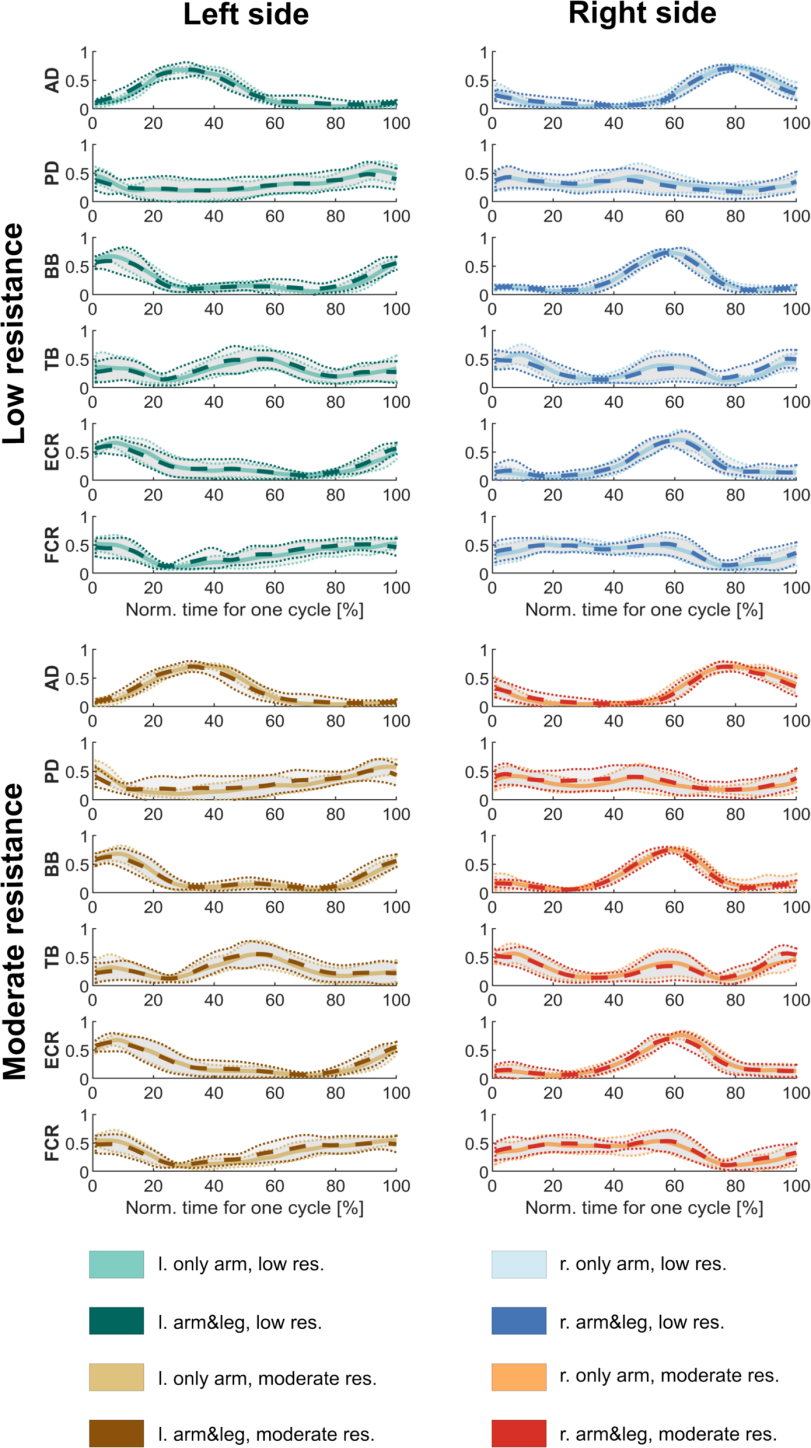
Mean muscle activities (across cycles and across participants) recorded from six arm muscles for each setup. EMG signals (with standard deviations) were compared between arm&leg (dashed curves) and only-arm cycling (continuous curves) modes

The average and standard deviations of time-normalized EMG envelopes (across participants) of the recorded arm muscle activities from the two cycling modes (only-arm and arm&leg) are presented in Fig. 2.

For all the recorded muscles, the EMG envelopes from both cycling modes (only-arm and arm&leg) were very similar, both on the left and right sides. Only minor differences can be observed in the graphs comparing the two cases. The EMGs from PD, TB, ECR and FCR presented considerable standard deviations, and the EMG envelopes obtained from the two cycling modes overlapped. Notably, the EMGs from the other two muscles with small standard deviations (anterior deltoid (AD) and biceps brachii (BB)) also overlapped when comparing only-arm and arm&leg curves. These observations are supported by the high correlation coefficients (higher than 0.85) obtained when comparing the EMG envelopes from the different cycling modes (Table 1).

**Table 1:** Correlation coefficients of averaged (across participants) EMG envelopes for each muscle with respect to the cycling mode (only-arm and arm&leg) based on the Spearman rank test

	AD	PD	BB	TB	ECR	FCR
left low	0.965	0.946	0.890	0.936	0.982	0.898
left, mod	0.958	0.919	0.987	0.946	0.993	0.957
right, low	0.939	0.862	0.955	0.885	0.958	0.908
right, mod	0.960	0.941	0.986	0.941	0.973	0.974

### Evaluation of VAF values

**Fig. 3:**
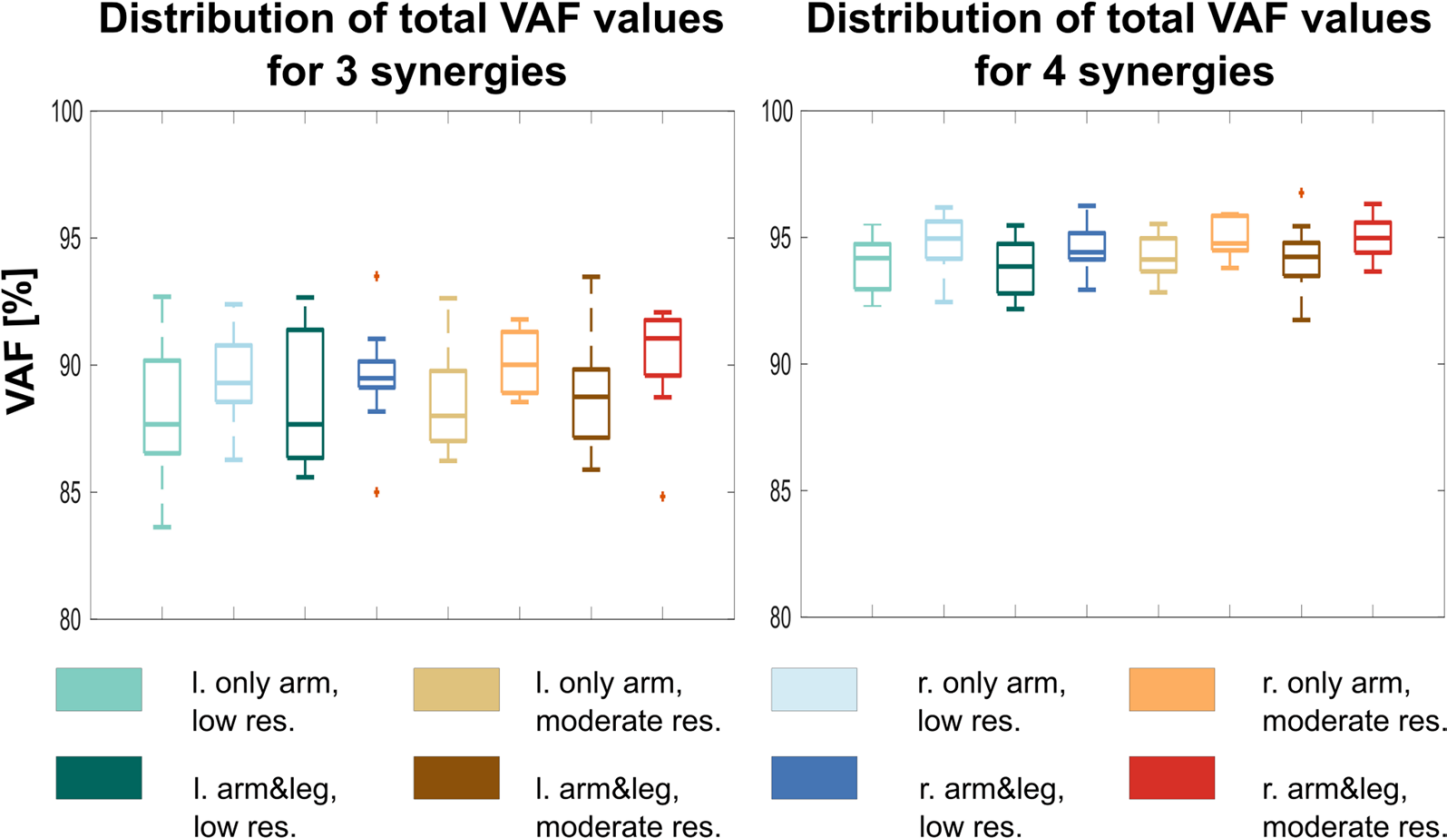
The distribution (among participants) of total VAF values were presented for different cycling setups for three and four synergies. The VAF by four synergies explained more than 90% of the variance of the EMG envelopes. The setups were as follows: 1) only arm with low resistance, left side; 2) only arm with low resistance, right side; 3) arm and leg cycling with low resistance, left side; 4) arm and leg cycling with low resistance, right side; 5) only arm with moderate resistance, left side; 6) only arm with moderate resistance, right side; 7) arm and leg cycling with moderate resistance, left side; 8) arm and leg cycling with moderate resistance, right side

The total VAF values were calculated for each cycling setups. Using three synergies, total VAF values were below 90% for most settings (minimum of total VAF was 84% and maximum was 94%). Using four synergies, total VAF values ranged from 92 to 97%. None of the participants required five synergies to achieve the acceptance criteria (to exceed 90% VAF). Since four synergies accounted for more than 90% of the variance in muscle activation in all settings, for all participants, four synergy vectors were applied for further analysis. The distribution (among the participants) of total VAF values were presented in boxplot diagrams for the different cycling setups for 3 and 4 Synergies (Fig. 3).

The median of total VAF values was higher for 4 Synergies in all setups. Considering four synergies, VAF values did not differ meaningfully comparing cycling modes or resistance levels.

### Comparison of muscle synergies components

**Fig. 4:**
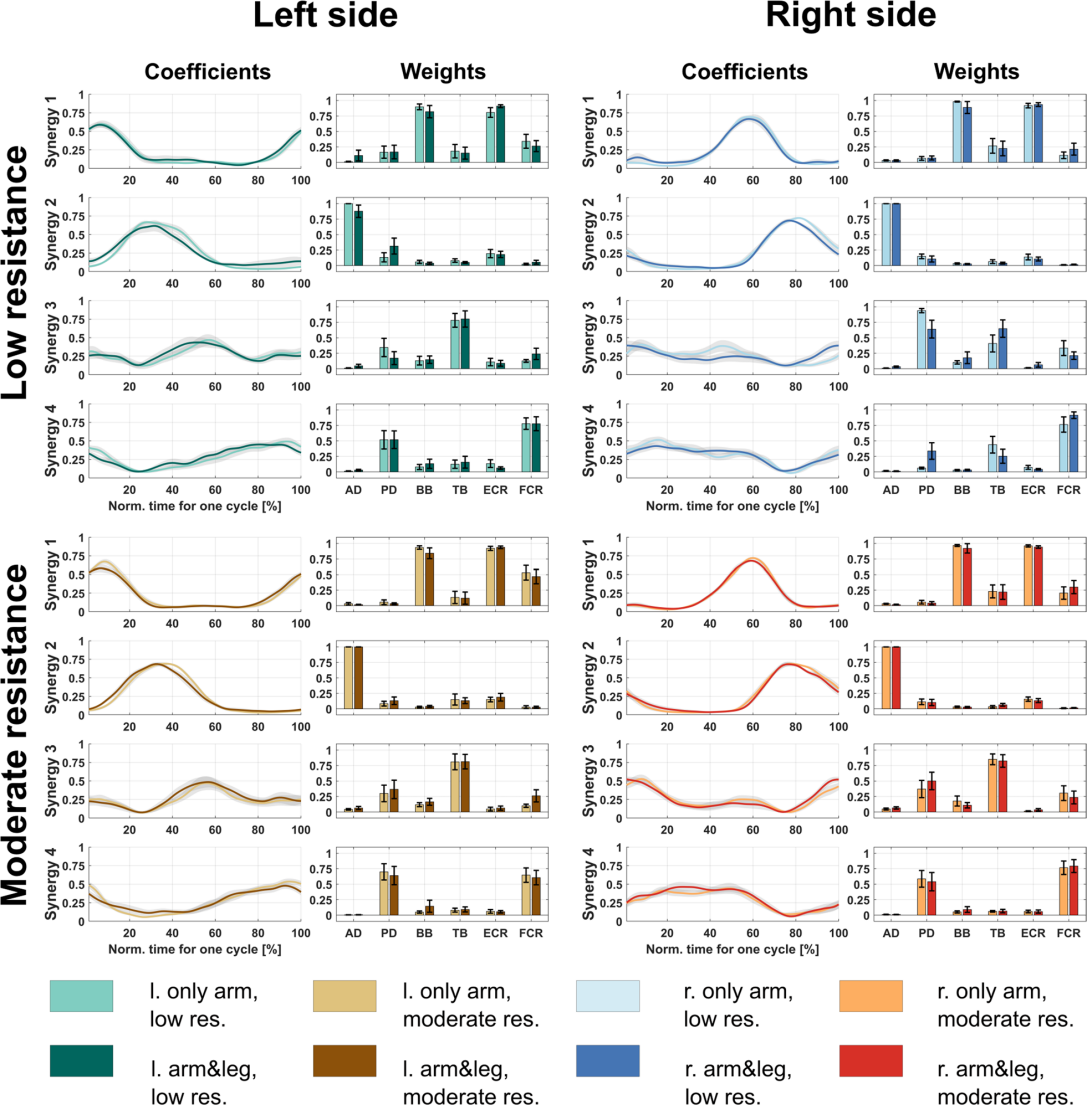
Averaged activation coefficients and synergy vectors obtained in eight setups. The activation coefficients are presented as a function of normalized time. The shaded area represent the standard errors. The bar diagrams show the average and standard errors (across participants, n = 10) of muscle weights in each synergy vector

The results of the synergy calculations are shown in Fig. 4. The average and standard error of the synergy weights (W) and coefficients (H) across the participants are illustrated.

We compared the synergy vectors in pairs according to the following: we compared the cycling setups between low and moderate resistance and between the “only arm” and “arm&leg” cycling modes in level of activation coefficients and in synergy weights.

With respect to the activation coefficients, the results of similarity analysis the rho values based on Spearman rank test was presented in the Table 2. The detailed analysis with p-values and Cohen’s d effect size is reported in the Appendix Table 1. There was statistically significant high correlation between the cycling modes and resistance levels (*p* < 0.05, d > 0.8). Only in one case, in arm&leg cycling mode, the left arm was not significantly high in the correlation (rho = 0.46, *p* = 0.09, d = 0.6) between the two resistances.

**Table 2:** Similarity of activation coefficients based on Spearman rank test. The setups were as follows: resistance—low (low) or moderate (mod), cycling mode—only-arm or arm&leg, and side—left or right side. Boldface represents significant differences

	Averaged (across participants) correlation of activation coefficients							
	Resistance				Cycling mode			
	left, only arm	left, arm &leg	right, only arm	right, arm &leg	left, low	left, mod	right, low	right, mod
S1	0.84	0.82	0.51	0.78	0.76	0.90	0.59	0.81
S2	0.89	0.74	0.63	0.79	0.80	0.79	0.58	0.71
S3	0.75	0.70	0.93	0.92	0.68	0.74	0.83	0.90
S4	0.79	**0.46**	0.97	0.95	0.65	0.71	0.91	0.94

The comparison of the activation coefficients using the SPM method did not reveal statistically significant differences between the setups. There was no significant difference between the resistances nor between the cycling modes (Appendix Fig. 1).

Regarding synergy weights, the cosine similarity was presented between the cycling combinations, in the Table 3. The synergy weights between the low and moderate level of resistance were similar for left side, in two cycling modes, and for the right side for arm&leg cycling mode (cosine similarity was higher than 0.83). Only for third Synergy weights, in the right side, only arm cycling mode was the cosine similarity below 0.8 (0.76), which was the similarity threshold in other studies [33, 34].

**Table 3:** Cosine similarity of synergy weights. The setups were as follows: resistance—low (low) or moderate (mod), cycling mode—only-arm or arm&leg, and side—left or right side. Boldface represents significant differences

	Cosine similarity of averaged (across participants) synergy weights							
	Resistance				Cycling mode			
	left, only arm	left, arm &leg	right, only arm	right, arm &leg	left, low	left, mod	right, low	right, mod
S1	1.00	0.96	0.99	1.00	0.99	0.99	0.99	0.99
S2	0.99	0.97	1.00	1.00	0.96	0.98	1.00	1.00
S3	0.97	0.89	**0.76**	0.97	0.93	0.95	0.92	0.99
S4	0.98	0.92	0.83	0.95	0.97	0.95	0.93	0.99

Complementary with these findings, the comparison of synergy weights, using Friedman test, is presented in the Appendix Table 2. Since we performed multiple comparisons (we examined the four cycling combinations in pairs, separately on the right and left sides), the corrected significance level due to the Holm-Bonferroni correction is alpha = 0.006. Based on the correction, the Conover post-hoc test also did not show significant differences between the cycling combinations.

## Discussion

In our present study, simultaneous arm and leg cycling and only-arm cycling exercises were performed by able bodied persons. Arm muscle synergies were computed using nonnegative matrix factorization, and the synergies obtained in simultaneous arm&leg cycling and in only-arm cycling were investigated.

### Effect of leg cycling on arm muscle coordination

The main aim of this study was to investigate whether leg cycling, added simultaneously to arm cycling, affects arm muscle synergies in able-bodied people. Our results show that there were no significant differences in the muscle synergy vectors or in the activation coefficients between the 2 cycling modes (only-arm cycling and arm&leg cycling). This suggests that the arm muscle coordination is invariant to leg cycling, and the central nervous system employs the same arm muscle coordination strategy, when lower limb activity is added to the examined arm cycling movement. The neural coordination of only-arm cycling is not necessarily influenced by the movement of the body parts, such as the lower limbs, whose main role is locomotion.

This investigation of arm muscle coordination helps to discern how upper-limb motor control is influenced by lower-limb activity and provides insights into sensorimotor integration and has implications in neurorehabilitation. Understanding how leg cycling affects arm coordination is important for designing rehabilitation protocols, including hybrid exercises (e.g. for people with spinal cord injury (SCI) or for stroke survivors).

The literature on the effects of lower limb cycling on upper limb movements and muscle activation is relatively limited compared with that on the effects of arm cycling on leg muscle activation. One study [18] investigated synergies both for arm cycling and leg cycling but not for simultaneous cycling. Biomechanical and electrophysiological methods have been used to investigate the effects of lower limb cycling on upper limb kinematics and muscle activation, leading to various results [12, 35, 36]. Sakamoto investigated the effect of leg cycling cadence on arm cycling cadence and reported that, during simultaneous arm and leg cycling, arm cycling cadence significantly decreased during an instant change in leg cycling cadence. This suggests a neural interaction of the central pattern generator systems that control arm cycling and leg movements.

These studies, which investigated the effect of lower limb cycling on arm muscle activation, considered either kinematic or electrophysiological properties of the studied systems and motor tasks. A novelty of our study is that we applied the muscle synergy approach to investigate whether lower limb cycling influences upper limb muscle coordination. Our results did not support the assumption that lower limb cycling affects upper limb muscle coordination during arm cycling. Specifically, no effect was observed when the lower limb cycling conditions remained unchanged. The reason may be that the lower limb cycling under unchanged conditions was not a sufficient stimulus to evoke changes in upper limb coordination despite interlimb neural coupling. Cycling is a rhythmic periodical movement that may be performed quite independently of other motor tasks. Functional couplings between the cervical and lumbar spinal segments are likely task-dependent and addition of rhythmic motor tasks may not induce a reorganization of arm muscle coordination.

### Effect of crank resistance on muscle coordination

The secondary aim of our study was to investigate whether different arm crank resistances require different muscle coordination in terms of muscle synergies. The results revealed that synergies were not affected by crank resistance in either cycling mode or in either the dominant or non-dominant arm. A previous study showed that variance profiles of muscle activation in arm cranking was not affected by crank resistance [37]. The amplitude of muscle activation (assessed by EMG) increases with higher loads, but the activation profile does not necessarily alter.

The EMG signal consists of a combination of activation coefficients and synergy weights, so the magnitude of the activation coefficient is not directly depended on the EMG amplitude. Thus, it is possible that there is a slight difference between the two resistances in both the weights and the activation coefficients, but it is not significant.

Similar result was reported in former research on reaching arm movements suggesting that synergy amplitude coefficients showed small variability with changes of load [38].

The cycling task in our study used low loads which may not provide sufficient stimulus to alter synergies, but higher loads might have caused changes in synergies. Chaytor et al. [39] studied the effect of workload on EMG amplitudes during arm cycling and showed a linear relationship between EMG amplitude of the examined muscles and the power output. Only the slope of the linear relation differed among muscles. However, muscle coordination was not investigated. Esmaeili et al. [40] found high degrees of similarity among muscle synergies in leg cycling across various load conditions, and demonstrated that different mechanical conditions use the same motor control strategies for cycling. If muscle synergies are shared across load conditions in lower-limb cycling, it is reasonable to assume that similar properties may apply to upper-limb cycling.

### Effects of cycling mode and crank resistance on VAF values

We assumed that cycling with four limbs is more challenging than cycling only with the arms, and this is reflected in the number of arm muscle synergies that dominantly account for the arm muscle activation variance (VAF). In a recent study, it was shown that for walking tasks, a greater number of synergies are used if the task is more difficult. In particular, leg muscle synergies were assessed and compared during walking on a taped line on the floor and on a 6-cm wide, 2-cm high beam [41]. A greater number of muscle synergies were recruited during beam walking, which was more challenging than tape walking for both young and older participants.

In our study if adding leg cycling to arm cycling leads to a more difficult motor task, that might be associated with a larger number of arm muscle synergies. However, we found that the same number of synergies are able to generate muscle activities with VAF higher than 90% (Fig. 3). Arm cycling performed simultaneously with leg cycling may require more attention [12], but from an arm muscle coordination point of view, it does not seem to be more demanding than arm cycling alone.

### Implications for rehabilitation

The common neural control of lower limb and upper limb movements has implications for the rehabilitation of patients with SCI. Dietz [42] posited consequences for rehabilitation of task-dependent neuronal linkage of cervical and thoracic–lumbar propriospinal circuits controlling leg and arm movements. Simultaneous arm and leg cycling is an excellent candidate for rehabilitation therapy for patients with incomplete SCI. The combination of arm and leg cycling in rehabilitation training protocols may result in higher oxygen uptake than cycling by only two limbs. This is a desired goal of cycling exercises [43, 44]. From the aspect of motor control, our present study suggests that arm muscle coordination is preserved while the CNS controls the cyclist’s leg movements in addition to arm movement. Arm cycling control can be maintained when training setups are changing. Our previous study revealed that arm configuration variance during arm cranking was not affected by changes in crank resistance [37]. Our present study suggests that arm muscle coordination in terms of muscle synergies is not affected by crank resistance either. Furthermore, muscle coordination in arm cycling does not depend on additional training setups, such as simultaneous leg cycling.

Here, we provide insights into the relationship between leg cycling and arm cycling control in able-bodied, healthy individuals. In other studies, arm cycling exercises have been used in combination with functional electrical stimulation-assisted leg cycling training (hybrid cycling) in individuals with SCI [45]. Further studies are needed to investigate arm cycling control during hybrid cycling in people with neurological disorders.

### Limitations

The gender body structure may differ [46–48], but for reasons of homogeneity, only female participants’ movements were investigated in the present study. Although the number of participants is small, this group is homogeneous in terms of age and gender, therefore we assume that our analysis is relevant. To support this, we applied several quantitative comparison methods -based on the literature- to exclude the random results. These methods were correlation analysis, SPM, cosine similarity and Friedman-test. However, our results may not be generalizable to a wider population. Further studies should investigate more patients to build on the present study.

The designed protocol considered that the performance of the arm&leg cycling exercise demanded focused attention from the participants. Therefore, they had a short familiarization time to practice the exercise. Our experience indicated that a randomized ordering of the crank resistance levels would have required significantly longer practicing time, which we aimed to limit in order to avoid the long-term skill acquisition and overlearning of the motor task. In this sense, we had to find a practical compromise to execute our measurement protocol while minimizing the risk of overlearning.

To discard measurement artifacts, cycle selection criteria were applied. The cycle selection process might hide some differences in motor control between conditions. At the same time, high standard deviations resulting from artifacts can also obscure characteristic parameters. We attempted to set the criterion in such a way as to filter out deviant signals while retaining the characteristics of the given cycling setup. The remaining amount of data (on average more than 60 cycles per condition) is still enough for our study based on the recommendations of Turpin et al. [24].

In this study, one flexor‒extensor muscle pair was selected for the shoulder, elbow and wrist joints, the most important and strongest flexors and extensors of these joints. Extracting four synergies when recording six muscles seems to be a limited dimension reduction. However, we believe that comparing synergies and VAF values for movement tasks performed under different setups provides useful information about the possible effects of these setups on the central control of the movement. Even if the considered number of muscles is small and the number of muscles is a limitation of the study, we believe that our exploratory study gives insights into important characteristics of arm cycling coordination during only arm cycling and simultaneous arm and leg cycling motor tasks.

In the present work, we focused on investigating the effect of the legs on the arms in two different load levels on the arms. Further, investigation of the influence of lower limb movement on the upper limbs should include a protocol in which the resistance for upper limb exercise is constant while varying the lower limb cycling resistance conditions.

Future studies might focus on the effect of arm cycling on leg muscle synergies as well.

## Conclusion

Our results suggest that the central nervous system applies similar control strategies for arm cycling regardless of the inclusion of leg cycling. The neuromuscular system tends to preserve synergy structure in arm muscles when lower limb cycling is added to arm cycling and when arm crank resistance is added. These observations could be considered in the formulation of rehabilitation protocols.

## Figures and Tables

**Fig. 5:**
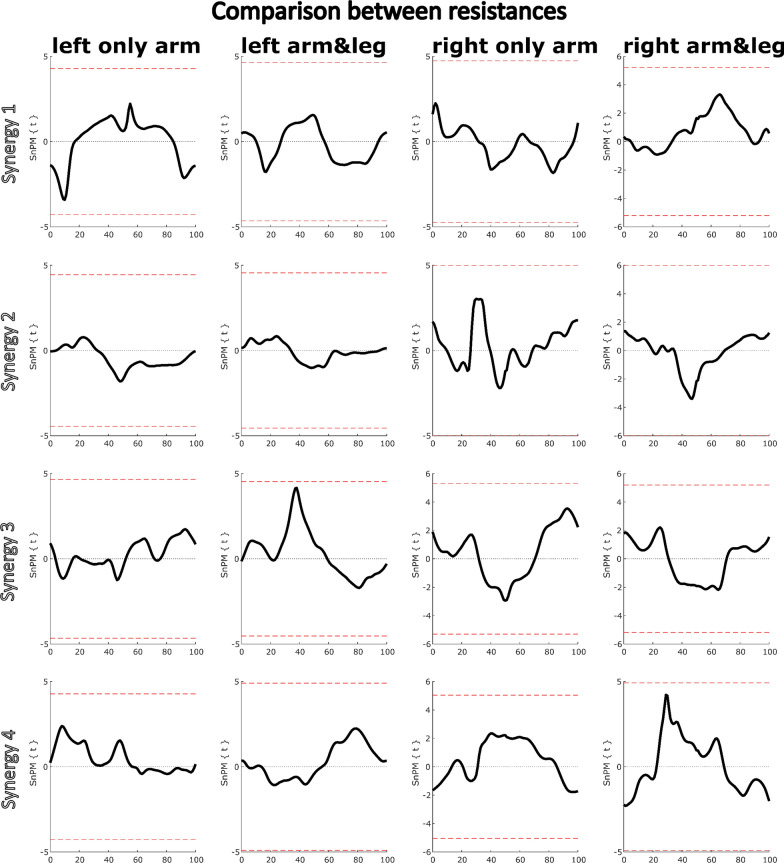
Statistical comparision between low and moderate resistances is based on Statistical Parametric Mapping (SPM). This method calculates the differences between the activation coefficients (black line). Points where the SPM curve exceeds the significance threshold (indicated by the red dashed line) are interpreted as Statistical significant differences. In the present analysis, no significant differences were observed between the activation coefficients with respect to the resistances

**Fig. 6:**
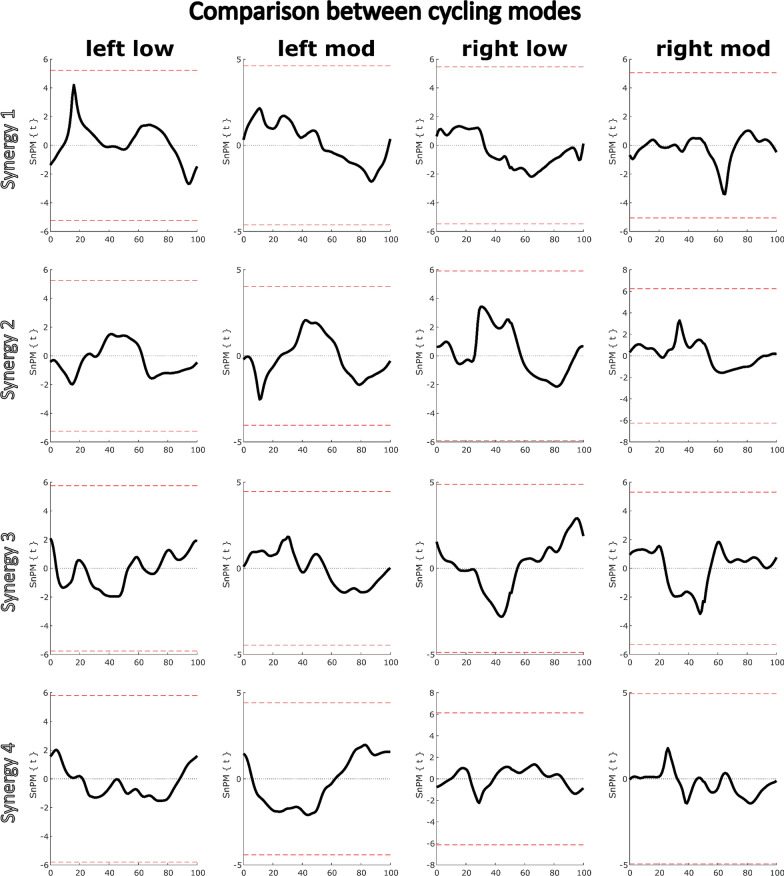
Statistical comparision between arm&leg and only arm cycling modes is based on Statistical Parametric Mapping (SPM). This method calculates the differences between the activation coefficients (black line). Points where the SPM curve exceeds the significance threshold (indicated by the red dashed line) are interpreted as Statistically significant differences. In the present analysis, no significant differences were observed between the activation coefficients with respect to the cycling modes

## Data Availability

Raw and analyzed data are available from the corresponding author on reasonable request.

## Appendix

See (Tables

**Table 4:** Detailed correlation analysis of activation coefficients

		Resistance				Cycling mode			
		left, only arm	left, arm &leg	right, nly arm	right, arm &leg	left, low	left, mod	right, low	right, mod
S1	rho	0.84	0.82	0.51	0.78	0.76	0.90	0.59	0.81
	p	0.00	0.00	0.02	0.00	0.00	0.00	0.03	0.00
	d	2.29	2.10	0.91	1.38	2.14	2.29	0.83	2.66
S2	rho	0.89	0.74	0.63	0.79	0.80	0.79	0.58	0.71
	p	0.00	0.03	0.01	0.00	0.02	0.01	0.00	0.00
	d	1.42	0.81	1.15	1.43	0.90	0.96	1.50	1.20
S3	rho	0.75	0.70	0.93	0.92	0.68	0.74	0.83	0.90
	p	0.00	0.01	0.00	0.00	0.01	0.00	0.00	0.00
	d	1.24	1.00	2.58	2.66	1.08	1.31	2.02	2.86
S4	rho	0.79	0.46	0.97	0.95	0.65	0.71	0.91	0.94
	p	0.00	0.09	0.00	0.00	0.00	0.02	0.00	0.00
	d	1.35	0.60	4.71	5.01	1.36	0.90	3.71	3.16

The “rho” value means the results of Spearman rank correlation, the p is significance level (calculated by the Fisher-z transformation), and the is the Cohen’s d effect size

4,

**Table 5:** Comparison of the differences between cycling combinations in level of synergy weights. The setups were as follows: resistance—low (low) or moderate (mod), cycling mode—only-arm or arm&leg, and side—left or right side. Friedman test was used with Conover post-hoc test for the comparison of the weights of each muscle in each synergy vector, with a *p* < 0.006 Holmes-Bonferroni corrected significance level

		Resistance						Cycling mode			
		left, AR	left, AL	right, AR	right, AL			left, low	left, mod	right, low	right, mod
synergy 1	AntDel	0.26	0.14	0.85	0.26	synergy 1	AntDel	0.40	0.64	0.71	0.19
	PosDel	0.60	0.66	0.48	0.66		PosDel	0.11	0.54	0.79	1.00
	BicBra	0.53	0.85	0.85	0.53		BicBra	0.70	0.67	0.89	0.34
	TriBra	0.82	0.20	0.53	0.86		TriBra	0.31	0.62	0.79	0.86
	ExCarRa	0.49	0.49	1.00	0.64		ExCarRa	0.93	0.93	0.58	0.93
	FlCarRa	0.29	0.02	0.50	0.63		FlCarRa	0.27	0.89	0.29	0.39
synergy 2	AntDel	0.83	0.83	1.00	1.00	synergy 2	AntDel	0.51	0.51	1.00	1.00
	PosDel	0.72	0.66	0.53	0.48		PosDel	0.33	0.86	0.66	0.59
	BicBra	0.29	0.72	0.86	0.86		BicBra	0.93	0.19	0.54	0.54
	TriBra	0.31	0.27	0.36	0.93		TriBra	0.20	0.41	1.00	0.31
	ExCarRa	0.86	0.86	0.11	0.37		ExCarRa	0.32	0.53	0.93	0.42
	FlCarRa	0.59	0.59	0.48	0.48		FlCarRa	0.79	0.79	0.13	0.93
synergy 3	AntDel	0.05	0.96	0.06	0.11	synergy 3	AntDel	0.07	0.92	0.18	0.29
	PosDel	0.11	0.02	0.01	0.58		PosDel	0.09	0.03	0.32	0.18
	BicBra	0.54	0.86	0.93	0.73		BicBra	0.86	0.54	1.00	0.80
	TriBra	0.87	0.12	0.02	0.74		TriBra	0.27	0.51	0.35	0.30
	ExCarRa	0.20	0.78	0.47	0.06		ExCarRa	0.15	0.93	0.36	0.78
	FlCarRa	0.13	0.42	0.69	0.96		FlCarRa	0.86	0.37	0.79	0.47
synergy 4	AntDel	0.54	0.79	0.48	0.38	synergy 4	AntDel	0.38	0.60	0.48	0.38
	PosDel	0.57	0.08	0.02	0.02		PosDel	0.68	0.06	0.68	0.64
	BicBra	0.59	0.25	0.42	0.21		BicBra	0.18	0.72	1.00	0.65
	TriBra	0.78	0.38	0.16	0.03		TriBra	0.85	0.34	0.96	0.41
	ExCarRa	0.06	0.47	1.00	0.79		ExCarRa	0.79	0.37	0.47	0.65
	FlCarRa	0.48	0.02	0.67	0.48		FlCarRa	0.48	0.33	0.45	0.63

^5^) and (Figs. 5, 6).

## Abbreviations

CNS: Central nervous system
EMG: Electromyogram
NNMF: Non-negative matrix factorization
VAF: Variance accounted for
SPM: Statistical Parametric Mapping
AD: Anterior Deltoid
PD: Posterior Deltoid
BB: Biceps Brachii
TB: Triceps Brachii
ECR: Extensor Carpi Radialis
FCR: Flexor Carpi Radialis
SCI: Spinal cord injury
